# Diagnostic efficacy of real-time PCR for ocular cytomegalovirus infections

**DOI:** 10.1007/s00417-018-4111-9

**Published:** 2018-08-27

**Authors:** Dai Miyazaki, Daisuke Shimizu, Yumiko Shimizu, Yoshitsugu Inoue, Tomoyuki Inoue, Shiro Higaki, Mayumi Ueta, Sunao Sugita, Real-time PCR for ocular cytomegalovirus infection study group, Dai Miyazaki, Daisuke Shimizu, Yumiko Shimizu, Yoshitsugu Inoue, Tomoyuki Inoue, Yuichi Ohashi, Shiro Higaki, Yoshikazu Shimomura, Mayumi Ueta, Shigeru Kinoshita, Sunao Sugita, Manabu Mochizuki

**Affiliations:** 1 Division of Ophthalmology and Visual Science, Faculty of Medicine, Tottori University, 36-1 Nishi-cho, Yonago, Tottori 683-8504 Japan; 2 Department of Ophthalmology, Ehime University School of Medicine, Matsuyama, Japan; 3 Department of Ophthalmology, Kindai University Faculty of Medicine, Osaka-Sayama, Japan; 4 Department of Frontier Medical Science and Technology for Ophthalmology, Kyoto Prefectural University of Medicine, Kyoto, Japan; 5 Laboratory for Retinal Regeneration, Center for Developmental Biology, RIKEN, Kobe, Japan

**Keywords:** Cytomegalovirus, Corneal endotheliitis, Real-time PCR, Anterior uveitis, Retinitis

## Abstract

**Purpose:**

The aim of this study is to determine the efficacy of quantitative real-time PCR (qPCR) and clinical characteristics to diagnose ocular cytomegalovirus (CMV) infections.

**Methods:**

The technical factors were assessed by the outcomes of the qPCR assay at five institutions in Japan using the WHO International Standard of cytomegalovirus. The clinical factors were assessed by examining the aqueous humor samples of 197 eyes of 197 consecutive patients suspected of CMV using the receiver operating characteristics (ROCs).

**Results:**

All of the institutions had excellent detection efficacy, although the copy number ranged from 0.82 to 4.66 copies/IU. In the clinical samples, CMV was detected in 51 eyes, and the amount of CMV DNA was significantly higher for CMV retinitis. In corneal diseases, the amount of CMV DNA was significantly associated with frequency of recurrences and IOP elevations. The sensitivity and specificity of qPCR for the diagnosis was 90.0 and 98.7%, respectively. For the corneal and anterior uveitis types of CMV diseases, the area under the curve (AUC) of qPCR was 0.95 and 0.96, followed by frequency of recurrences with AUC of 0.89 and 0.82, and IOP elevations with AUC of 0.78 and 0.76. Unclassified cytomegalovirus detection, which did not meet diagnostic criteria of CMV corneal endotheliitis, anterior uveitis, or retinitis, was 4.6%, and it was significantly associated with corneal diseases and history of corneal transplantation.

**Conclusions:**

qPCR with standardization is specific and accurate; however, the inclusion and knowledge of the clinical characteristics improve the diagnostic efficacy.

## Introduction

Ocular infections caused by cytomegalovirus (CMV) are well-known to lead to CMV retinitis in immune-compromised patients. In immune-competent patients, CMV corneal endotheliitis and CMV uveitis have been diagnosed mainly in elderly individuals. The clinical signs of CMV can also be observed in non-HIV patients with retinitis [1].

CMV infections of the anterior segment are relatively rare and often misdiagnosed. CMV corneal endotheliitis can present as bullous keratopathy or keratitis after years of recurrent episodes. CMV uveitis can present as Fuchs heterochromic iridocyclitis or Posner-Schlossman syndrome with recurrences of elevations of the intraocular pressures (IOPs) [2]. For CMV corneal endotheliitis, the hallmark signs of the disease are endothelial cell loss, coin-shaped lesions, IOP elevations, and owl’s eye appearance of the lesions. An earlier study showed that these signs had a highly positive predictive value of 90.9% for the diagnosis of CMV [3]. For CMV uveitis, a relapsing inflammation is a well-known and characteristic sign, and it may be accompanied by endothelial cell loss. However, the diagnosis of CMV infection is difficult when made by only the clinical signs and symptoms.

PCR has been established as the standard diagnostic method for diagnosing systemic CMV infections achieving a sensitivity of 80.1% and specificity of 93% for blood samples [4]. For the diagnosis of ocular CMV infection, the amount of aqueous sample is limited, and the efficacy of quantitative real-time PCR (qPCR) has not been adequately validated. In addition, a CMV infection of the eye in immune-competent patients is relatively rare and is mainly observed in the Asian population.

Thus, the purpose of this study was to determine the efficacy of qPCR and clinical characteristics that will allow clinicians to detect and diagnose ocular CMV infections in individuals living in Japan [2] [5]. To accomplish this, we first evaluated the efficacy of CMV qPCR at five major ophthalmological institutions throughout Japan. We determined institutional or clinical factors which had affected the diagnostic efficacy of ocular CMV infection using a CMV standard and aqueous humor samples from consecutive cases of suspected ocular CMV infections. The results showed that qPCR of CMV is a very good test with excellent efficacy for diagnosing ocular CMV infections. However, knowledge of the clinical characteristics and unclassified CMV detection improves the efficacy of the diagnosis.

## Materials and methods

### Patients

The medical records of 197 eyes of 197 consecutive patients examined over a 13-year period were studied. All of these patients were suspected of having an ocular CMV infection or required an exclusion diagnosis. These patients had keratitis including corneal endothelial inflammation, the corneal disease type, or anterior uveitis or retinitis which did not respond to conventional therapy including topical steroids. All of the cases had undergone qPCR of the aqueous humor for CMV and differential diagnosis of viral infections.

One hundred thirteen of the 197 patients were men. The mean age of all the patients was 63.2 ± 15.6 years. All of the patients were examined at the Tottori University Medical Hospital between November 2005 and May 2017. A total of 290 measurements were made to diagnose the recurrent inflammations or to assess the therapeutic efficacy of antiviral treatment.

All procedures were performed in accordance with the ethical standards of the Institutional Review Board of Tottori University, Tottori, Japan, and the procedures conformed to the 1964 Helsinki declaration and its later amendments of comparable ethical standards. An informed consent was obtained prior to the procedures from all of the participants.

To compare the reliability of the qPCR measurements at the different institutions, the 1st WHO International Standard for Human Cytomegalovirus for Nucleic Acid Amplification Techniques was used. This standard sample contained 5 × 10^6^ international units (IUs) of CMV. One IU corresponded to one copy of the CMV genome. The 95% detection limit was calculated using international standard DNA diluted to 10^3^, 10^2^, 10^1^, 10^0^, 10^−1^, and the concentration that achieved 95% positivity by qPCR assessment was determined to be 98 IU as calculated by probit regression (*N* = 20/dilution) [6]. This indicated that when 20 samples of 98 IU of CMV was assayed, 19 samples would be diagnosed as positive for CMV.

To determine the copy numbers of the CMV DNA in the aqueous humor samples of the consecutive case series, DNA was extracted from the aqueous humor with QIAamp DNA mini kit (Qiagen, Hilden, Germany). The glycoprotein B gene of the CMV was amplified with the LightCycler (Roche, Basel, Switzerland) [7] [8] (Table 1). A standard curve was generated with known dilutions of cloned DNA amplicons. Samples with less than the 95% detection limit were taken to be negative [6].

**Table 1:** Settings for real-time PCR and primers at different institutions

Facility	DNA extraction	Real-time PCR	CMV amplicon	Primers and probe
A	EZ1 Advanced XL EZ1 Virus Mini (Qiagen)	Light cycler 2.0 (Roche)	IE-1	CATGAAGGTCTTTGCCCAGTAC GGCCAAAGTGTAGGCTACAATAG Probe TGGCCCGTAGGTCATCCACACTAGG
B	QIAamp Viral RNA Mini (Qiagen)	ABI 7000 (Applied Biosystems)	DNA polymerase	GCTGACGCGTTTGGTCATC ACGATTCACGGAGCACCAG Probe FAM-TCGGCGGATCACCACGTTCG-TAMRA
C	QIAamp DNA Mini (Qiagen)	StepOnePlus (Applied Biosystems)	DNA polymerase	GCTGACGCGTTTGGTCATC ACGATTCACGGAGCACCAG Probe FAM-TCGGCGGATCACCACGTTCG-TAMRA
D	DNA Extract All (Applied Biosystems)	StepOnePlus (Applied Biosystems)		Pa034534000_s1 Applied biosystems
E	QIAamp DNA Mini Kit (Qiagen)	Light cycler 2.0 (Roche)	Glycoprotein B	AAGTACCCCTATCGCGTGTG ATGATGCCCTCRTCCARGTC

For differential diagnosis of infections by herpes simplex virus (HSV) and varicella-zoster virus (VZV), the aqueous humor sample was assayed for HSV and VZV by qPCR as described in detail [9]. Primers detecting both HSV-1 and HSV-2 were used for the HSV assays.

### Diagnosis and clinical parameters for assessment of ocular CMV infection

The final diagnosis of CMV infection was made by the course of the disease process and confirmed by the detection of the DNA of CMV by conventional PCR or antigenemia [2, 5, 10–13]. An ocular CMV infection was classified as CMV corneal endotheliitis, CMV anterior uveitis, or CMV retinitis.

CMV corneal endotheliitis was diagnosed when the following criteria, (a and (b or c)), were met [5]:

- Positive for CMV DNA in the aqueous humor, and negative for the DNA of HSV and VZV.
- Corneal endotheliitis with coin-shaped lesion or linear keratic precipitates (KP).
- Corneal endotheliitis with localized edema with two of the following were present; recurrent anterior uveitis or ocular hypertension or corneal endothelial loss.

CMV anterior uveitis was diagnosed when (a, d, and e) were met [11, 13].

d. Recurrent anterior uveitis without retinitis or posterior segment inflammation.
e. Responsiveness to anti-viral drugs for CMV.

CMV retinitis was diagnosed when (a and f) were met [14].

f. Characteristic necrotizing retinitis.

Unclassified CMV detection was present when CMV DNA was positive in the aqueous humor of cases which did not require anti-CMV drug therapy and were not caused by an ocular CMV infection by the above diagnosis criteria as described [15].

To assess the diagnostic efficacy of the clinical parameters for CMV infection, the number of recurrences, IOP elevations of > 20 mmHg without glaucoma medication, loss of corneal endothelial cells, history of keratoplasty, and presence of coin-shaped keratic precipitates were determined. Corneal endothelial cell loss was defined as < 1000 cells/mm^2^ or less than 500 cells/mm^2^ in the contralateral eye [7].

### Statistical analyses

Data are presented as means ± standard deviation. For bilateral cases, the findings of the more severely affected eye were used for the statistically analyses. ANOVA and post-hoc tests were used to determine whether the differences between the groups were statistically significant. Multivariate logistic regression analysis was used to compute the odds ratio (OR). Clinical signs including IOP elevation, corneal endothelial cell loss, and previous history of corneal transplantation were analyzed as dichotomic variable (present or absent). The frequency of recurrences was coded as 0, 1, 2, and ≥ 3. To evaluate the diagnostic efficacy of qPCR, the receiver operating characteristic (ROC) regression analysis was used with adjustments for repeated measurements and clinical parameters. Statistical analyses were conducted with Stata 15 (Stata Corp, College Station, Tx). A *P* < 0.05 was considered significant.

## Results

### Institution-dependent technical effects on reliability of qPCR

We first analyzed whether institutional factors may have affected the outcome of qPCR of CMV DNA. Five major institutions that routinely perform qPCR for diagnosing ocular CMV infections participated in this collaborative study. The WHO International Standard of CMV were sent to each institution, and the CMV DNA copy numbers were determined using the institutions’ own protocol of DNA extraction and qPCR. Technicians belonging to each facility extracted the DNA from the standard using their routine protocol and quantified the amount of CMV DNA using different qPCR equipment, primers, and cloned templates (Table 1). The standard containing 5 × 10^6^ IU of CMV was serially diluted to 10^4^ to 10^−1^ IU in 10-fold steps, and the DNA was extracted. The extracted DNA was assayed for the copy numbers of DNA.

The results showed that the International Unit Standard was correlated with the copy number (ρ = 0.94, *P* = 0.0000); however, the calculated copy numbers were significantly different for the same amount of CMV standard. For 1 unit of the International Standard, the calculated copy number ranged from 0.82 to 4.66 copies (Table 2), and the mean copy number was 1.92 copies with 95% confidence intervals of 1.63 to 2.22 (*P* < 0.001). This indicated that exact evaluations require standardization by the International Units, and the use of facility-dependent copy number may overestimate the amount of CMV DNA by up to fourfold.

**Table 2:** Efficacy of CMV real-time PCR evaluated by WHO CMV standard

	Copy/IU	95% confidence interval	*p* value	Area under curve
				Cut-off (95% probability detection)
A	1.20	1.08–1.33	0.000	1.0
B	1.26	1.13–1.39	0.000	1.0
C	1.68	1.56–1.81	0.000	1.0
D	4.66	4.54–4.79	0.000	1.0
E	0.82	0.70–0.95	0.000	1.0

We also assessed whether the results of qPCR for CMV differed at the different institutions using ROC analysis. The WHO CMV standard diluted up to the sensitivity limits was evaluated for presence or absence of CMV DNA (Table 2). When the limit of detection for clinical diagnosis was defined as the amount of DNA needed to diagnose the sample as positive with 95% probability [6], all the facilities showed an AUC of 1.0 and had reliable and consistent performances.

### Association of CMV DNA with clinical signs and disease type

To assess the clinical factors which were associated with the amount of CMV DNA in the eye, we evaluated the characteristics of the CMV ocular infection and other herpes family viruses. The diseases were classified into three types; corneal disease type (keratitis involving endothelial inflammation), anterior uveitis, and retinitis (Table 3). The anterior uveitis type of disease made up 54.3% (107 cases) of all cases. Of these, 17.8% (19 cases) were caused by CMV-associated anterior uveitis which was the most frequent, and it was significantly more frequent than HSV (*P* = 0.006, proportional test). The corneal disease type was diagnosed in 69 eyes (35.0%), and of these, CMV was the most frequent cause at 34.8%. This was followed by infections of HSV (20.3%). For the retinitis type of diseases, 4 of the 21 cases were caused by CMV. Collectively, CMV was the most frequent cause of intraocular viral infections.

**Table 3:** Causative viral pathogen and disease type

	Corneal endotheliitis type	Anterior uveitis type	Retinitis type
Cytomegalovirus	34.8% (24 eyes)	17.8% (19 eyes)	19.1% (4 eyes)
Herpes simplex virus	20.3% (14 eyes)	5.6% (6 eyes)*	4.8% (1 eyes)
Varicella zoster virus	2.9% (2 eyes)**	9.3% (10 eyes)	4.8% (1 eyes)
Total (eyes)	69	107	21

*P = 0.006; **P = 0.0000, proportional test

**Fig. 1:**
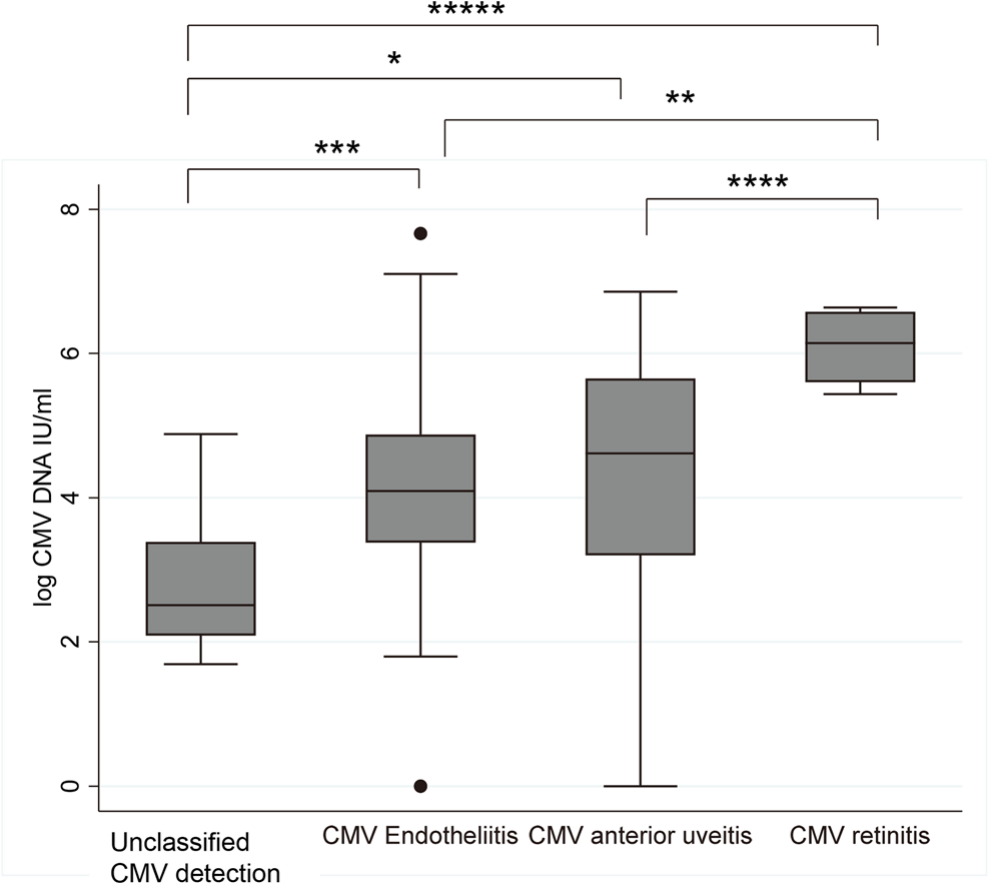
Disease type-dependent cytomegalovirus DNA amounts at the first presentation. CMV retinitis showed significantly higher cytomegalovirus DNA than other types of CMV infections. CMV DNA amount of shedding was significantly lower than those of ocular CMV infections. *P = 0.01; **P = 0.005; ***P = 0.002; ****P = 0.001; *****P = 0.000

At the initial presentation, the average logarithm of the IU of CMV was 4.1 ± 2.2 log IUs for the CMV anterior uveitis. The average CMV IUs for the endotheliitis type of CMV was 4.2 ± 1.8 log IU, and it was 6.1 ± 0.6 log IU for the retinitis type of CMV (Fig. 1). CMV retinitis had significantly higher IUs than the other types of CMV infections, and an increase of anterior (keratitis) to posterior (retinitis type) was observed.

We next assessed how the CMV DNA may be associated with the clinical characteristics using logistic regression analysis of all the cases with or without CMV infections (Table 4). CMV DNA was significantly associated with the frequency of recurrences and IOP elevations in the corneal and anterior uveitis types of diseases. In contrast, significant associations of CMV DNA with corneal endothelial loss were only observed for the anterior uveitis type.

**Table 4:** Association of CMV DNA with clinical characteristics by logistic regression analysis

	Corneal disease type			Anterior uveitis type			Retinitis type		
	OR	95% CI	*P* value	OR	95% CI	*P* value	OR	95% CI	*P* value
Frequency of recurrences	2.2	1.5–3.3	0.000	1.5	1.2–1.9	0.002	1		NS
IOP elevations	1.6	1.2–2.0	0.000	1.6	1.1–2.3	0.009	1.3	0.8–2.0	NS
Corneal endothelial cell loss	1	0.8–1.3	NS	1.4	1.1–1.8	0.002			NS

*OR* odds ratio (per log CMV IU/ml) after age adjustment

### Diagnostic efficacy of clinical characteristics and CMV DNA for ocular CMV infections

We then determined the disease type-dependent diagnostic efficacy of qPCR at the first visit (Table 5). The overall sensitivity and specificity of qPCR was 89.4 and 94.0%, respectively. The negative predictive value was excellent at 96.6%, respectively. The sensitivity and specificity profiles were similar for the corneal and the anterior uveitis types of diseases.

**Table 5:** Sensitivity and specificity profile of CMV real-time PCR

	Overall		Corneal disease type		Uveitis type	
	95% CI		95% CI		95% CI	
Sensitivity	89.4%	76.6–95.6%	91.7%	70.9–98.0%	84.2%	59.7–95.1%
Specificity	94.0%	88.8–96.9%	88.9%	75.4–95.4%	95.5%	88.3–98.3%
Positive predictive value	82.4%	69.2–90.7%	81.5%	61.6–92.3%	80.0%	56.2–93.3%
Negative predictive value	96.6%	92.0–98.6%	95.2%	82.2–98.9%	96.6%	89.7–98.9%
False positive rate	17.7%	9.3–30.8%	18.5%	7.7–38.4%	20.0%	7.4–43.8%
False negative rate	3.4%	1.4–8.0%	4.8%	1.1–17.8%	3.5%	1.1–10.3%

**Fig. 2:**
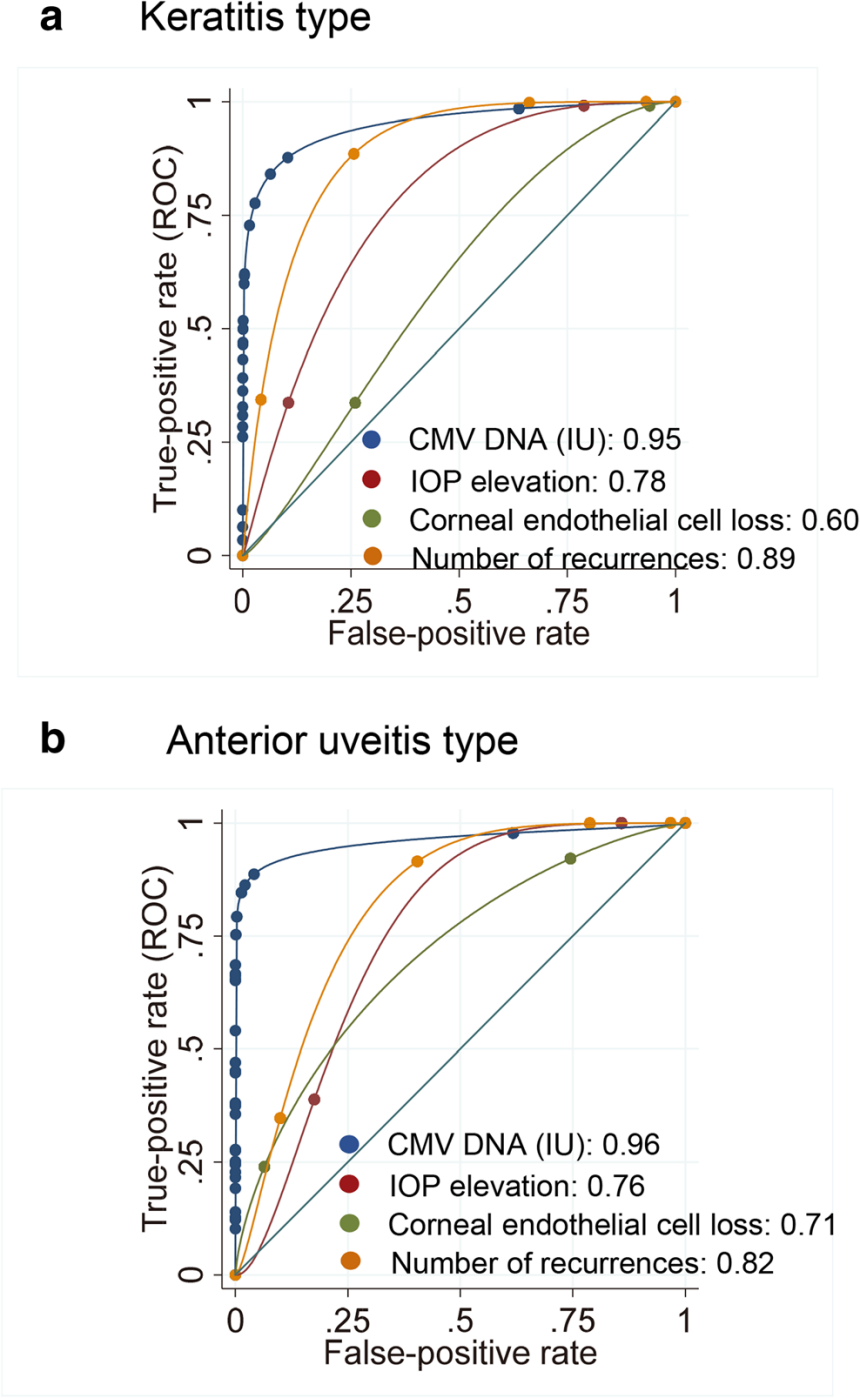
Receiver operating characteristics regression analysis of cytomegalovirus real-time PCR of aqueous humor and clinical signs for corneal disease type and uveitis type of disease. Cytomegalovirus real-time PCR showed excellent area under the curve (AUC) of 0.98. **a** For the clinical signs of corneal disease type, the number of recurrences and IOP elevations had good AUCs of 0.89 and 0.78, respectively. **b** For the anterior uveitis type of disease, the number of recurrences had a good AUC of 0.82, followed by IOP elevation (AUC 0.76)

Next, we compared the diagnostic efficacy of qPCR and the clinical parameters. To assess the efficacy of the diagnosis, the area under the curve (AUC) was calculated using ROC regression after adjustments for the clinical parameters (Fig. 2). The overall AUC of qPCR was 0.98. For the corneal type of diseases, the AUC of qPCR was 0.95 followed by number of recurrences (AUC 0.89), and IOP elevation (AUC 0.78). For the uveitis type of disease, the AUC for qPCR was 0.96 followed by the number of recurrences (AUC 0.82) and IOP elevation (AUC 0.76). These findings indicate that the number of recurrences is the most important clinical sign. For the CMV retinitis cases, the four CMV cases were diagnosed correctly using CMV qPCR, and the AUC was 1.0.

### Factors associated with unclassified CMV detection

We next assessed factors which were associated with unclassified CMV detection, which did not meet criteria of ocular CMV detection and did not require anti-viral treatment. The unclassified CMV detection was low at 4.6% (nine eyes). The corneal disease type had higher rate of the detection (five eyes, 7.2%), and the anterior uveitis type had a frequency of 3.7% (four eyes).

When logistic regression analysis was performed to assess any associations with the unclassified CMV detection, the corneal disease type and history of corneal transplantation had significant odds ratio of 8.8 (*P* = 0.004) and 8.4 (*P* = 0.006, after age adjustment), respectively. No other clinical characteristic, including the IOP elevation, corneal endothelial cell loss, number of recurrences, and infection of HSV or VZV, was significantly associated with the unclassified CMV detection.

## Discussion

The results showed that the AUC for the diagnostic efficacy of CMV qPCR for aqueous samples was 0.98 for ocular CMV infections, and no other single clinical sign matched this high AUC. This indicated that qPCR for CMV is the most efficient single diagnostic method to detect ocular CMV infections, and its use allows an accurate diagnosis at the first visit when any type of ocular CMV infection is suspected.

Clinicians generally rely on the clinical signs for their diagnosis. We showed that the factors which significantly affected the diagnosis of ocular CMV infection was dependent on the disease type. The most useful clinical signs for the diagnosis were the frequency of recurrences of the corneal and anterior uveitis types of CMV infections. CMV corneal endotheliitis can be diagnosed very effectively with the clinical characteristics of the frequency of recurrences.

Suspected anterior segment viral infections, including the corneal disease types and anterior uveitis types, were associated with CMV, HSV, and VZV. We showed that CMV was the most frequent cause of these diseases. The results indicated that the exclusions of HSV and VZV were very important for the diagnosis of CMV infections. For the exclusion of HSV infections, a history of dendritic lesions or recurring stromal keratitis should be considered. A history of herpes zoster ophthalmicus highly suggests the presence of ocular VZV infection [16] [17]. Thus, the exclusion of HSV and VZV may also be achieved by the history, and DNA detection is not required. This information will benefit clinicians in the differential diagnosis of ocular CMV infections effectively. In addition, all of these Herpes virus family members are well known to shed spontaneously from healthy tissues and can be detected in diseased eye. However, we did not observe co-detection of CMV and HSV or VZV in the aqueous samples.

We showed that CMV qPCR has high specificity for diagnosing ocular CMV infections. However, we observed cases with unclassified CMV detection. In elderly subjects, more than one-half were seropositive for CMV, and the frequency of shedding of CMV in body fluids including urine and serum was approximately 7% [15]. There remain clear whether unclassified CMV detection was caused by shedding. In our ocular inflammatory disease cases, the corneal disease type was most significantly associated with the unclassified CMV detection, and its main characteristic was a history of corneal transplantation. Under these conditions, we need to be aware that presence of CMV DNA may not require treatment.

The differences in the disease type may reflect where the CMV is latently infected before causing an active infection. Thus, the identification of the tissue of CMV detection without requirement of treatment is most likely the latently infected tissue. We found a significant association of unclassified CMV detection and the corneal disease type and previous history of corneal transplantation. This suggests that the probable tissue where CMV resides latently is the cornea, and we have shown that CMV can effectively replicate in the corneal endothelial cells [8].

In CMV retinitis, the amount of CMV DNA was significantly higher than in CMV corneal endotheliitis or anterior uveitis (Fig. 1). Generally, CMV retinitis is associated with systemic CMV infections, and the amount of CMV DNA in the blood is known to be a significant sign for poor prognosis and increased mortality [18] [19]. Transmission to the retina is considered to occur by transmission from the blood through the retinal vascular endothelium [20]. CMV retinitis is generally observed in immune compromised patients, and the reduced anti-viral responses may permit unrestricted viral proliferation. This may explain the higher amount of CMV DNA in CMV retinitis patients, and why presumable latency or unclassified detection was not observed for the retinitis type of CMV disease.

In the anterior uveitis type of CMV disease, the AUC for the number of recurrences and IOP elevations was lower than that for the corneal disease type. Such differences may be caused by strain differences of the CMV [21]. Currently, it remains unclear whether the endotheliitis type and the anterior uveitis type of CMV are caused by different CMV strains. However, Oka et al. reported no significant difference in the amount of viral load or viral protein profiles for these two types of diseases [22].

The quantitative aspects of CMV qPCR have not been well appreciated for the aqueous humor. Some researchers have concluded that a positive or negative detection of the DNA of CMV was sufficient information. However, we conclude that the viral load of CMV is also very important information for clinicians to determine the treatment protocol as is well known for systemic CMV infections [19]. For example, the viral load of CMV was significantly associated with the endothelial cell loss and glaucoma medications [2] [7]. Thus, a higher viral load will predict refractoriness or advancement of the stage of CMV infection. When treating anterior segment inflammations due to CMV, anti-viral drugs are used. After anti-CMV viral drugs are applied, the amount of CMV decreases, and the level generally becomes undetectable [23]. However, the clinical signs of endotheliitis or uveitis, and the IOP elevation can promptly resolve before the aqueous CMV amount becomes completely negative. A decrease in the viral load in the presence of low levels in the aqueous humor suggests a need of prolonged use of anti-viral drugs. However, the decision on whether to continue the use of antivirals is very difficult to make without tracking the viral load. In addition, the resistance to ganciclovir occurs in up to 10% of the cases when anti-viral treatment duration becomes longer in systemic infections [19]. Tracking of the amount of viral DNA not responding to the antiviral treatment will become an important sign to consider drug-resistant infections.

To detect the presence or absence of CMV, the qPCR facilities had equally efficient diagnostic efficacy. This was somewhat unexpected because the five facilities used different primers, reagents, and equipment. Importantly, the copy numbers by each institutional assay were different by up to fourfold for the same sample. This can be due to different target and amplification protocols, or DNA standards of copy numbers. In our hands, the amplification efficacy of different qPCR methods for CMV did not appear to cause significant changes of the copy numbers. Indeed, incorrect amplification is easily noticed by technician and by trouble shooting. However, problematic quality of DNA standard for copy number calculations is very difficult to be recognized. Although this can be tested by limiting dilution of standard to one copy of DNA, maintaining this high sensitivity is difficult for routine laboratory work. During trouble shooting processes, we noticed a decay or improper preparation of the DNA standards that can lead to overestimations of the template DNA concentration. This can cause significantly increased calculated copy numbers. This further supports the importance of validated DNA standards.

Thus, we propose the use of standardized international unit, which theoretically corresponds to one copy of genome. Generally, anterior segment CMV infections are rare, and the pooling of the data and comparisons of case series was required to accurately determine the etiology of the disease and the therapeutic strategy. Thus, reporting the viral loads in IUs can facilitate more accurate assessments of such trials, and efficient integration of data by meta-analysis will help determine the efficacy.

There are several limitations in our study. The efficacy of qPCR for clinical samples and incidence of ocular CMV infection were assessed using case series which were mainly referred to us for diagnosis and may not represent the exact incidence. In addition, the results of a non-selected population might differ. After this assessment of performance of qPCR, the protocol of PCR in each facility may have been updated to further improve efficacy of PCR for quality control purpose.

For the current study, the sensitivity of CMV qPCR was 98 IU as the limit of detection which is higher than the theoretical limit of detection of PCR (three copies) [6]. However, when 98 IU was used as the cut off value, no difference in the efficacy of qPCR was observed in the different institutions (Table 2). In addition, patients with less than cut off were not required for anti-CMV treatment.

Sensitivity of qPCR for ocular CMV infections was very high. However, CMV DNA was not always detected at the first visit, and some cases required repeated examinations of CMV DNA for the final diagnosis. Clinicians need to be aware that qPCR becomes negative when inflammations are not intense.

In conclusion, qPCR is a very effective diagnostic test; however, the CMV DNA amount depends on the clinical characteristics. Thus, determining the clinical characteristics will facilitate a more accurate diagnosis than simply relying on costly CMV qPCR testing.

## References

[1] Pathanapitoon K, Tesavibul N, Choopong P, Boonsopon S, Kongyai N, Ausayakhun S, Kunavisarut P, Rothova A (2013). Clinical manifestations of cytomegalovirus-associated posterior uveitis and panuveitis in patients without human immunodeficiency virus infection. JAMA Ophthalmol.

[2] Megaw R, Agarwal PK (2017). Posner-Schlossman syndrome. Surv Ophthalmol.

[3] Hwang YS, Shen CR, Chang SH, Lai CC, Liu CL, Chen KJ, Lin KK, Chen TL, Hsiao CH (2011). The validity of clinical feature profiles for cytomegaloviral anterior segment infection. Graefes Arch Clin Exp Ophthalmol.

[4] Boeckh M, Nichols WG (2004). The impact of cytomegalovirus serostatus of donor and recipient before hematopoietic stem cell transplantation in the era of antiviral prophylaxis and preemptive therapy. Blood.

[5] Koizumi N, Inatomi T, Suzuki T, Shiraishi A, Ohashi Y, Kandori M, Miyazaki D, Inoue Y, Soma T, Nishida K, Takase H, Sugita S, Mochizuki M, Kinoshita S, Japan Corneal Endotheliitis Study G (2015) Clinical features and management of cytomegalovirus corneal endotheliitis: analysis of 106 cases from the Japan corneal endotheliitis study. Br J Ophthalmol 99: 54–58 10.1136/bjophthalmol-2013-304625 PMC4283688 25075122

[6] Bustin SA, Benes V, Garson JA, Hellemans J, Huggett J, Kubista M, Mueller R, Nolan T, Pfaffl MW, Shipley GL, Vandesompele J, Wittwer CT (2009). The MIQE guidelines: minimum information for publication of quantitative real-time PCR experiments. Clin Chem.

[7] Kandori M, Miyazaki D, Yakura K, Komatsu N, Touge C, Ishikura R, Inoue Y (2013). Relationship between the number of cytomegalovirus in anterior chamber and severity of anterior segment inflammation. Jpn J Ophthalmol.

[8] Miyazaki D, Uotani R, Inoue M, Haruki T, Shimizu Y, Yakura K, Yamagami S, Suzutani T, Hosogai M, Isomura H, Inoue Y (2017). Corneal endothelial cells activate innate and acquired arm of anti-viral responses after cytomegalovirus infection. Exp Eye Res.

[9] Kakimaru-Hasegawa A, Kuo CH, Komatsu N, Komatsu K, Miyazaki D, Inoue Y (2008). Clinical application of real-time polymerase chain reaction for diagnosis of herpetic diseases of the anterior segment of the eye. Jpn J Ophthalmol.

[10] van Boxtel LA, van der Lelij A, van der Meer J, Los LI (2007). Cytomegalovirus as a cause of anterior uveitis in immunocompetent patients. Ophthalmology.

[11] Chee SP, Bacsal K, Jap A, Se-Thoe SY, Cheng CL, Tan BH (2008). Clinical features of cytomegalovirus anterior uveitis in immunocompetent patients. Am J Ophthalmol.

[12] Jeon S, Lee WK, Lee Y, Lee DG, Lee JW (2012). Risk factors for cytomegalovirus retinitis in patients with cytomegalovirus viremia after hematopoietic stem cell transplantation. Ophthalmology.

[13] Mochizuki M, Sugita S, Kamoi K, Takase H (2017). A new era of uveitis: impact of polymerase chain reaction in intraocular inflammatory diseases. Jpn J Ophthalmol.

[14] Jabs DA, Van Natta ML, Thorne JE, Weinberg DV, Meredith TA, Kuppermann BD, Sepkowitz K, Li HK, Studies of ocular complications of ARG (2004) Course of cytomegalovirus retinitis in the era of highly active antiretroviral therapy: 2 Second eye involvement and retinal detachment Ophthalmology 111: 2232–2239 10.1016/j.ophtha.2004.05.028 15582079

[15] Cannon MJ, Hyde TB, Schmid DS (2011). Review of cytomegalovirus shedding in bodily fluids and relevance to congenital cytomegalovirus infection. Rev Med Virol.

[16] Tran KD, Falcone MM, Choi DS, Goldhardt R, Karp CL, Davis JL, Galor A (2016). Epidemiology of herpes zoster Ophthalmicus: recurrence and chronicity. Ophthalmology.

[17] Inata K, Miyazaki D, Uotani R, Shimizu D, Miyake A, Shimizu Y, Inoue Y (2018). Effectiveness of real-time PCR for diagnosis and prognosis of varicella-zoster virus keratitis. Jpn J Ophthalmol.

[18] Jabs DA, Martin BK, Forman MS, Ricks MO, Cytomegalovirus R, Viral Resistance Research G (2005). Cytomegalovirus (CMV) blood DNA load, CMV retinitis progression, and occurrence of resistant CMV in patients with CMV retinitis. J Infect Dis.

[19] Jabs DA (2011). Cytomegalovirus retinitis and the acquired immunodeficiency syndrome—bench to bedside: LXVII Edward Jackson memorial lecture. Am J Ophthalmol.

[20] Rao NA, Zhang J, Ishimoto S (1998). Role of retinal vascular endothelial cells in development of CMV retinitis. Trans Am Ophthalmol Soc.

[21] Shimizu Daisuke, Miyazaki Dai, Shimizu Yumiko, Hosogai Mayumi, Kosugi Isao, Inoue Yoshitsugu (2018). Infection of endotheliotropic human cytomegalovirus of trabecular meshwork cells. Japanese Journal of Ophthalmology.

[22] Oka N, Suzuki T, Inoue T, Kobayashi T, Ohashi Y (2015). Polymorphisms in cytomegalovirus genotype in immunocompetent patients with corneal endotheliitis or iridocyclitis. J Med Virol.

[23] Koizumi N, Miyazaki D, Inoue T, Ohtani F, Kandori-Inoue M, Inatomi T, Sotozono C, Nakagawa H, Horikiri T, Ueta M, Nakamura T, Inoue Y, Ohashi Y, Kinoshita S (2017). The effect of topical application of 0.15% ganciclovir gel on cytomegalovirus corneal endotheliitis. Br J Ophthalmol.

